# Placebo effects of caffeine on sport performance: a systematic review and meta-analysis

**DOI:** 10.1080/15502783.2026.2747142

**Published:** 2026-10-20

**Authors:** Philip Hurst, Janina Hanssen, Felipe Marticorena, Lieke Schiphof-Godart, Hiago L. R. Souza, Moacir Marocolo, Bryan Saunders

**Affiliations:** a Department of Sport & Exercise, Canterbury Christ Church University, Canterbury, United Kingdom; b Applied Physiology and Nutrition Research Group – School of Physical Education and Sport and Faculdade de Medicina FMUSP, Universidade de São Paulo, São Paulo, Brazil; c Center of Lifestyle Medicine, Faculdade de Medicina FMUSP, Universidade de São Paulo, São Paulo, Brazil; d Erasmus University Rotterdam, Department of Medical Informatics, Rotterdam, The Netherlands; e Postgraduate Program in Biosciences and Human Health, Minas Gerais State University, Divinopolis, Minas Gerais, Brazil; f Federal University of Juiz de Fora, Department of Physiology, Juiz de Fora, Sao Paulo, Brazil

**Keywords:** Coffee, exercise, dietary supplements, motor control, psychobiology

## Abstract

**Background:**

A body of research has shown that deceptively administering a placebo described as caffeine can significantly improve sport performance. We aimed to synthesize and estimate the effect size of the deceptive administration of a placebo described as caffeine on sport performance outcomes (e.g. bench press, cycling time-trial, repeated sprint).

**Methods:**

We searched for studies sampling healthy adults from database creation to July 2026. The standardized mean difference (*d*) between placebo caffeine and baseline was calculated and Cochrane's risk of bias assessment was used.

**Results:**

Seventeen studies were included. Nine studies were judged to be of high-risk of bias and eight unclear. Random-effect models revealed a small-to-moderate effect of placebo caffeine benefiting performance (*d* = 0.49, 95% CI = 0.28 to 0.69) and meta-regressions indicated that pooled effects were more likely to be greater when researchers were blinded (b = 0.52 ± 0.24, 95% CI = 0.01 to 0.99) and in studies sampling more males (b = −0.93 ± 0.38, 95% CI = −1.68 to −0.19).

**Discussion:**

Data highlight that placebo effects of caffeine can significantly affect sport performance with a pooled effect estimate suggesting a small-to-moderate effect. However, as per the GRADE assessment, the overall quality of evidence was low. As such, given the high risk of bias across all studies and the variation in confidence interval, stronger research designs are needed to examine placebo effects of caffeine, whereby researchers are blinded to treatment administration and allocation concealment. No funding was received for the review and the protocol can be found here: doi: 10.17605/OSF.IO/W8KY6.

## Introduction

Placebo effects are a psychobiological response to a purported beneficial treatment [1]. A body of evidence has shown they can significantly improve sport performance [2]. Systematic reviews [3–5] and a meta-analysis [6] report that they can improve a number of outcomes, including speed [7,8], strength [9,10], and endurance [11,12]. Research into placebo effects on sport often examinesthe influence of ergogenic aids [2],with most investigating the effects of caffeine [3,13,14]. Caffeine is widely used by athletes of all ages and abilities [15–17]. In an umbrella review of 21 meta-analyses [18], caffeine benefited sport performance outcomes, with pooled effect estimates (Cohen's *d*) ranging from 0.16 to 0.63. Several mechanisms are purported to underpin effects, including acting as an adenosine receptor antagonist [15], enhancing free-fatty acid oxidation [19], and mobilization of intra-cellular calcium [20]. However, despite a large body of evidence highlighting both the beneficial effects and underpinning mechanisms of caffeine, some have argued that placebo effects play a key role in its effectiveness [3,13,14].

In a systematic review that included randomized, placebo-controlled trials of caffeine on sport performance, placebo effects were shown to exert a small effect [*d *= 0.09; 21]. However, placebo-controlled trials are not an ideal method for understanding placebo effects. That is, during these trials, participants are informed that they will receive either caffeine or a placebo, which can increase uncertainty about the treatment received. As placebo effects are largely dependent on expectation [22], uncertainty about whether caffeine was received, is likely to influence the occurrence of placebo effects [23]. To overcome this, placebo effect researchers deceptively administer placebos to participants and inform them that it is an active treatment [2]. To help provide greater understanding and comprehension of the magnitude, variability and conditions under which placebo effects are likely to influence the effectiveness of caffeine, a need exists in synthesizing research that deceptively administered placebos described as caffeine.

To address this knowledge gap, the objective of our systematic review and meta-analysis was to understand the deceptive administration of placebo described as caffeine^1^ on sport performance outcomes. We aimed to (1) conduct a systematic review and synthesize current evidence of placebo effects of caffeine on sport performance outcomes, (2) conduct a meta-analysis and determine the pooled effect of studies examining placebo effects of caffeine on sport performance, and (3) identify whether pooled effects varied in relation to different characteristics of each study.

## Methods

Following a predefined protocol [24], this study is reported in accordance with the guidelines provided by the Preferred Reporting Items for Systematic Review and Meta-analysis [25] statement. The full PRISMA checklist is available in Supplementary material.

### Eligibility criteria

Studies examining placebo effects of caffeine on sport performance outcomes were considered for inclusion. We applied a set of inclusion and exclusion criteria for both the systematic review and meta-analysis using the population, intervention, control, and outcome formulation [26]:

*Population*: Apparently healthy participants of both sexes, aged 18 or older

*Intervention*: Administration of a placebo deceptively described as caffeine

*Control*: A baseline comparison or no-treatment control group

*Outcome*: Sport performance (e.g. power output, speed, distance)

Given that placebo effects can only be inferred when a separate baseline or no-treatment control is included [1,4,5], we only included studies that met this criterion. Experimental, randomized, quasi-randomized, and non-randomized, as well as both crossover and parallel designs were considered for inclusion. Studies written in English, published, or in print, in a peer-reviewed journal were included.

### Information sources

We searched the following databases for eligible studies from their earliest records to July 2026: Ovid MEDLINE (from 1946), EMBASE (from 1971), Web of Science (from 1971), and EBSCO CINAHL (from 1982). To identify additional articles, we hand searched reference lists of all included studies and key reviews [e.g. 3,4,6,13]. Academics researching both placebo effects and caffeine were consulted for further studies. No dedicated grey-literature database or thesis/dissertation repository were systematically searched.

### Search strategy

The search strategy centered around three themes: (1) placebo effects, (2) caffeine, and (3) sport performance. In accordance with the Peer Review of Electronic Search Strategies [PRESS; 27] guidelines, the Ovid MEDLINE strategy was created with input from the project team and once finalized, was adapted to the syntax and subject headings of the other databases. All search strategies for each database can be found in Supplementary material.

### Selection process

Studies retrieved from searches were imported into the web app Rayyan [28] and duplicates automatically removed. Two investigators (PH, JH) searched for eligible studies independently in two stages. In the first stage, titles and abstracts were read, with those meeting eligibility criteria forwarded for full-text screening. As per the Cochrane Handbook [29], we were over-inclusive at this stage for selecting studies. A pilot test on 100 studies was conducted to clarify inclusion and exclusion criteria between reviewers. Intercoder agreement was assessed with Cohen's kappa (ƙ). When a lack of information was given in the title or abstract, full texts were sourced and disagreements between authors were resolved with a discussion or with the involvement of a third investigator (LSG). In stage two, the same investigators assessed the full text of each study for further classification. Investigators were blinded to each other's ratings, and pilot testing on 10 articles was conducted to ensure consistency. Disagreements were resolved in the same manner as stage one. For studies reporting the same data in multiple studies, data were extracted from both studies and authors identified the “main” data source [29]. The selection process is recorded in a PRISMA [25] diagram (Figure 1), which was created using the PRISMA2020 R package [30].

**Figure 1.**
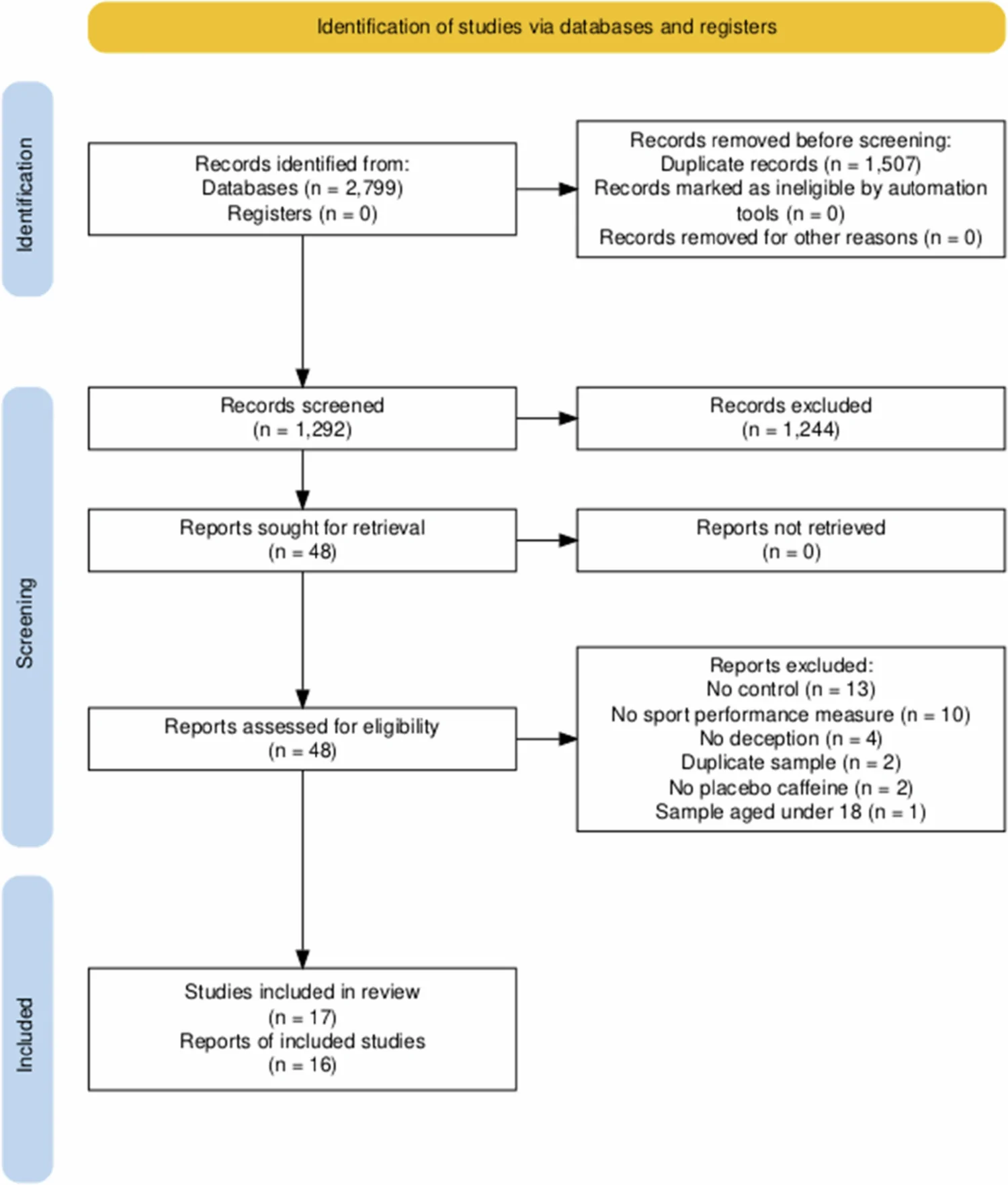
PRISMA flow chart of study selection.

### Data collection process

Three investigators (PH, HS, MM) extracted data independently, blinded and in duplicate for each study using a data extraction form (see Supplementary material). Extractions were compared between investigators, with disagreements resolved through discussion. Where consensus could not be reached, this was discussed and if necessary, we contacted study authors for clarifying information. Missing data, or data that was unclear, was requested from the corresponding study author via email. For authors who did not respond after two weeks, a follow-up email was sent, followed by a final reminder one-month later. If authors did not respond to requests, the study was considered for exclusion.

### Data items

Data extracted from each study included: study (i.e. lead author, year) and participant characteristics (e.g. description, sample size, sex, age), methodology (i.e. design, research and participant blind, control, familiarization trial, measurements), intervention (i.e. type, perceived dosage, timing, frequency, manipulation check, and who, how, where and when it was administered), outcomes (i.e. mean and standard deviations), and conclusions. Only data related to the placebo and baseline/control groups were extracted. The Template of Intervention Description and Replication [TiDier; 31] was used to extract intervention details from all studies.

Our interest was with the primary outcome of the placebo described as caffeine condition in comparison to baseline or no-treatment control. As studies often reported multiple outcomes, one primary outcome was allocated to each study to avoid duplication bias, with other outcomes identified as “secondary”. Using the guidelines outlined by the Cochrane Handbook for systematic reviews of interventions [29], the primary outcome most relevant to our research aim (i.e. placebo effects of caffeine on sport performance) was chosen.

All primary and secondary outcomes are reported in Table 1. Primary outcomes were categorized into the type of exercise it best represented (i.e. cycling, running, weightlifting) and duration (short [<1 min], moderate [1 to 10 min], and long [>10 min). Secondary outcome data included performance (e.g. mean power output), physiological (e.g. heart rate), biomechanical (e.g. step frequency), and psychological (ratings of perceived exertion [RPE]) measurements. Based on participant description, the sample for each study were categorized into trained or non-trained. Timing of the placebo administered prior to the measurement was categorized into (1) <1-min, (2) 1–20 min, and (3) >20 min, which represented the interval between placebo administration and performance testing. The <1-min category captured immediate testing, the 1–20-min a short-delay (including the shorter timing used for chewing gum), and the >20-min category captured for that associated with peak caffeine concentration. Finally, as placebo effect interventions often manipulate participant expectations about the intervention [e.g. oral presentation, providing scientific evidence, anecdotal reports; 32], we calculated the number of intervention components (e.g. verbal suggestions, scientific evidence, preconditioning procedure) that were reported based on the TiDieR guidelines [31].

**Table 1.** Study characteristics.

#	Author	Year	Sample description	Sample size (% female)	Age (years)	Research design	Measure	Type	Dose	Timing (min)	Primary	Secondary
1	Beedie	2006	Well-trained cyclists	6 (0)	30 ± 11	Cross-over	10-km cycling time-trial	Capsule	9.5 mg·kg^−1^	0	Mean power output	Lactate, HR, V̇O_2_
2	Foad	2008	Competitive cyclists	14 (0)	43 ± 7	Cross-over	40-km cycling time-trial	Capsule	5.0 mg·kg^−1^	0	Mean power output	Lactate, HR, V̇O_2_
3	Pollo study 1	2008	Healthy male volunteers	22 (0)	21.7 ± 2.3	Parallel	60% of 1-RM Leg-extension to failure	Decaffeinated coffee	High	15	Total work	RPE, number of extensions
4	Pollo study 2	2008	Healthy male volunteers	22 (0)	22.4 ± 3.1	Parallel	60% of 1-RM Leg-extension to failure	Decaffeinated coffee	High	15	Total work	RPE, number of extensions
5	Duncan	2009	Experienced resistance trained men	12 (0)	22.7 ± 6.0	Cross-over	60% of 1-RM Leg-extension to failure	Saline	3.0 mg·kg^−1^	60	Number of repetitions	HR, RPE, total weight lifted, systolic, and diastolic blood pressure
6	Piedimonte	2015	Healthy adults	30 (0)	21 ± 0.5	Parallel	60% of 1-RM finger flexion during 30-min	Decaffeinated coffee	NR	0	RPE	Electroencephalogram (EEG)
7	Brietzke	2017	Physically active individuals	9 (NR)	26.4 ± 4.8	Cross-over	Maximal incremental cycling test	Capsule	6.0 mg·kg^−1^	60	Time to exhaustion	RPE, peak power output
8	Costa	2019	Paralympic weightlifters	4 (0)	40.3 ± 10.0	Cross-over	Bench press throw	Capsule	6.0 mg·kg^−1^	60	Mean velocity	Mean velocity to peak, mean propulsive velocity, peak velocity
9	Hurst	2019	Well-trained middle-distance runners	11 (0)	25.2 ± 5.6	Cross-over	1000-m running time-trial	Saline	3.0 mg·kg^−1^	60	Time-to-complete	Hr, RPE
10	Shabir	2019	Soccer players	8 (0)	22 ± 5	Cross-over	20-m running at 95% V̇O_2_ max, Loughborough Intermittent Shuttle test	Capsule	3.0 mg·kg^−1^	60	Time to exhaustion	Time to complete test, RPE, HR, lactate, blood glucose, reaction time
11	Filip-Stachnik	2020	Resistance trained adults	13 (100)	23.0 ± 0.8	Cross-over	Bench press	Capsule	6.0 mg·kg^−1^	60	1RM	Reps to failure, time under tension, mean power output, peak power output, mean velocity, peak velocity
12	Rohloff	2022	Healthy runners	22 (0)	25.5 ± 8.4	Cross-over	4000-m running time-trial	Capsule	4.0 mg·kg^−1^	60	Time-to-complete	RPE
13	Campelo	2023	Trained adults	15 (0)	41 ± 4	Cross-over	Bench press at 40 and 80% 1RM	Capsule	NR	50	Number of repetitions	RPE
14	Ortiz-Sanchez	2024	Physically active individuals	18 (33)	19.7 ± 2.3	Cross-over	Bench press and back squat at 90% 1RM	Capsule	9.0 mg·kg^−1^	60	Mean power output	Mean velocity, velocity peak, mean acceleration, acceleration peak, peak power output, mean force, peak force, rate of force development, HR, heart rate variability
15	Valero	2024	Recreationally trained long-distance runners	13 (0)	38.5 ± 11.9	Cross-over	6-min running time-trial	Chewing gum	NR	20	Distance	Hr, RPE, step frequency, vertical oscillation and contact time
16	Agudo-Ortega	2025	Trained sprinters	13 (46)	22.8 ± 4.7	Cross-over	60-m sprint	Capsule	NR	50	Time-to complete	Standing triple jump distance
17	Valero	2026	Physical active young adults	25 (32)	22 ± 2	Cross-over	Bench press to failure at 75% 1RM	Capsule	3.0 mg·kg^−1^	60	Mean velocity	Countermovement jump, 15 s repeated jump test and bench press throw velocity at 25 and 50%1RM

Note: Data are mean ± standard deviation. NR = Not recorded,1RM = 1 repetition maximum, HR = heart rate, RPE = rating of perceived exertion, V̇O_2_ = oxygen consumption.

### Study risk of bias assessment

We used Cochrane's risk of bias assessment [29] to assess risk of bias for each study. Two reviewers (PH, FM) independently assessed each study for selection bias (sequence generation and allocation concealment), performance bias (participant and researcher bias), attrition bias (incomplete outcome data and dropouts), reporting bias (unreported findings), and other biases (e.g. publication bias, carry over effects). Assessments were classified as high (2), unclear (1) or low (0). An “unclear” rating was used when insufficient detail was given to confidently judge “low” or “high,” which could have included, for example, ambiguous, lack of or no information in the reporting of methods (e.g. allocation concealment not specified). Studies were then assigned an overall rating: high-quality study = all criteria classified as having a low risk of bias; unclear quality = at least one criterion rated as unclear; and low-quality = at least one criterion classified as high risk. Disagreements were resolved through discussion.

### Effect measures

The standardized mean difference (SMD) was calculated between baseline^2^ and placebo conditions. That is, we divided the mean difference between baseline and placebo by the pooled standard deviation and expressed this as Cohen's d (*d*). Effect sizes were interpreted as small (0.2), moderate (0.5), and large (0.8) [33]. Positive effects were indicative of improvements in the placebo condition compared to baseline, whereas negative indicated worsening.

## Synthesis methods

For our first study aim, we summarized and tabulated each study's characteristics and conclusions. For our second study aim, to determine the pooled effect of studies examining placebo effects of caffeine on sport performance, we conducted a meta-analysis using Comprehensive Meta-Analysis Software v4 (Bistat Inc., Englewood, NJ, USA) and evaluated the overall effectiveness of placebo versus baseline on the primary outcome for each study. In addition, following the guidance of Cumpston, Li [29], we conducted subgroup analyses when there were at least two studies examining a similar outcome. Beedie, Stuart [34] compared a placebo described as both 4.5 and 9.0 mg kg^−1^ of caffeine and we included data related to the latter in the analyses. Similarly, Costa, Galvão [35], Ortiz-Sanchez, Bravo-Sanchez [36] and Valero, Viudez [37] examined performance during increasing intensities (e.g. 50%, 60%, 70%) of 1 rep max (1RM), and we chose the greatest intensity for analyses. Given the large heterogeneity between each study in type of measurement (e.g. cycling time-trial, finger flexion strength), we used a random-effects model which was considered *α* priori [24]. The proportion of the variation in effect sizes that could be due to between-study variation was quantified using the *I* ^2^ statistic and interpreted as low (25%), moderate (50%), and high (75%) heterogeneity. The Cochran Q statistic was also used, with a *p* < 0.05 indicating the presence of heterogeneity [38]. Sensitivity analysis was conducted to examine the influence of individual studies (leave one out analysis) and study bias on meta-analyzed effects. Sensitivity analyses were only used when there were at least 10 studies in the meta-analysis [29]. 95% confidence intervals (95% CI) are reported alongside mean ± standard error and statistical analyses were two-tailed, with significance set at *p* < 0.05.

For our third study aim, to identify whether pooled effects varied in relation to different characteristics, moderators of the meta-analyzed effects were analyzed using a random effects meta-regression. Moderators were selected based on the availability of data across all studies, which included: type of exercise (i.e. cycling, running, weightlifting), duration of exercise (i.e. short, moderate, long), sample ability (i.e. trained, untrained), proportion of female participants, mean age of sample, study design (i.e. cross-over, parallel), if participants and researchers were blinded (i.e. yes, no), if randomization, familiarization and a separate control group were included (i.e. yes, no), type of placebo (e.g. decaffeinated coffee, saline, capsule), the perceived dose of caffeine, timing of administration (e.g. <1 min, 1 to 20 min, >20 min), whether actual caffeine was administered within the study (i.e. yes, no), the number of manipulations used in the intervention and the risk of bias score. For analyses that included less than 10-studies, we did not perform meta-regressions to minimize the risk of overfitting [29,39] and due to the small number of studies in each meta-analysis, we assessed moderators one at a time.

## Reporting bias assessment

We used Egger's regression test, with a *p* < 0.10 indicating the presence of publication bias [40]. Fail-safe numbers (FSN) were calculated, which represent the number of studies required to be included in the analysis that render the true prevalence as zero. When the FSN *N* is larger than the number of studies (*k*) that contribute to each analysis, publication bias is not considered [41]. Methods to detect funnel plot asymmetry were not used as they can be underpowered in meta-analyses of few trials of similar sample sizes [42] and assessments for publication bias were not conducted for analyses that included less than 10 studies [29].

## Certainty assessment

The certainty of evidence was assessed using the Grading of Recommendations, Assessment, Development, and Evaluations (GRADE) approach for the primary outcome analysis [43]. Certainty of evidence was judged on five domains: (1) risk of bias (e.g. high risk of bias across most studies), (2) inconsistency (e.g. high *I* ^2^), indirectness (e.g. studies assessing different populations of interest), (4) imprecision (e.g. large confidence intervals of pooled effects), and (5) publication bias (e.g. significant Egger's test). Each domain is rated from very low (i.e. little confidence in the effect estimate) to high (very confident that the true effect lies close to that of the estimate of effect). Two authors (PH, FM) rated each domain independently with discrepancies resolved through discussion.

## Results

### Study selection

A summary of the study selection process is summarized in the PRISMA flowchart (Figure 1). Our search identified 2799 records. After removing duplicates (*k* = 1507), we screened 1292 records, of which 1244 were excluded. Following this, we retrieved and assessed the full text of 48 reports for eligibility, of which 32 reports were excluded for the following reasons: 13 did not include a no-treatment control or baseline [44–56], 10 did not measure a sport performance outcome [57–66], 4 did not use a deceptive design [12,67–69], 2 were a duplicate sample [70,71], 2 did not administer a placebo described as caffeine [72,73], and 1 sampled under 18 year old athletes [74]. We therefore identified 17 studies (16 reports)^3^ for inclusion in the systematic review and meta-analysis. Intercoder agreement between reviewers was excellent for each stage of study selection (*ƙ* = 99%).

## Study characteristics

Characteristics of the 17 studies are shown in Table 1, and a summary of each individual study is presented in Supplementary material. Studies were published between 2006 and 2026. Most used a cross-over design (*k* = 14), which were pre-post-test (*k* = 10) or balanced placebo factorial design (*k* = 4), whereas for parallel design studies (*k* = 3), all were pre-post-test. Randomized designs and familiarization trials were conducted in 13 studies. Five studies compared a placebo described as caffeine alongside the actual administration of caffeine.

Two-hundred and fifty-seven participants (median age = 23.0 years,minimum to maximum = 19.7 to 43.0 years) were recruited across all studies, of which the majority were male (*n* = 210, 82%). Twelve studies included males only, one included females only and four included both sexes. Of the studies that included a mixed-sex sample, the average percentage of females was 40% (minimum to maximum: 32% to 47%). Studies sampled trained (*k =* 12) or non-trained (*k =* 5) participants.

Nine studies measured weightlifting performance, five running and three cycling. Ten primary outcomes were reported across all studies, with power output (*k* = 3) and time-to-complete (*k* = 3) most examined. Other primary outcomes included total work performed (*k =* 2), number of repetitions (*k =* 2), time-to-exhaustion (*k =* 2), 1 RM (*k* = 1), distance (*k* = 1), mean velocity (*k =* 2), and RPE (*k =* 1). A further 14 secondary outcomes were measured in the included studies, which were categorized as performance (i.e. time-under tension, reaction time, kinematics, and jump height), physiological (i.e. heart rate, lactate, oxygen consumption, blood pressure, EEG, and blood glucose), and psychological (i.e. expectations of caffeine, side effects of caffeine, and mood state).

For the placebo intervention, most studies administered a capsule (*k =* 11), with others using decaffeinated coffee (*k =* 3), saline solution (*k =* 2) or chewing gum (*k =* 1). Over half of studies administered the placebo > 20 min (*k =* 11) before experimental trials, with others administering it between 1- and 20-min (*k =* 3) and <1 min (*k =* 3). The perceived dose of caffeine varied between studies, with four studies stating low (≤3.0 mg kg^−1^), five moderate (4.0 to 6.0 mg·kg^−1^), and four large (>6.0 mg·kg^−1^). Four studies did not report the dose of caffeine.

Details of the placebo intervention for each study based on the TiDier checklist [31] can be found in Supplementary material. Studies reported 7 different methods in which to manipulate participant expectations about the placebo. Participants were provided with scientific evidence of the benefits of caffeine (*k =* 10) and verbally informed that it would improve their performance (*k =* 10). Three studies shared anecdotal evidence of the effectiveness of caffeine and one informed participants that caffeine used to be a prohibited performance enhancing substance. Information about the benefits of caffeine was delivered in a presentation (*k =* 2) or a pre-recorded video (*k =* 2). One study used a pre-conditioning procedure whereby the outcome in previous trials was surreptitiously improved. The median of methods in which to manipulate participant expectations about placebo caffeine was 2 (minimum to maximum = 0 to 5).

## Risk of bias

The quality of studies was poor, with 9 (53%) rated as high risk of bias, and 8 (47%) unclear. Allocation concealment was reported as high risk in over half of the studies (*k* = 9; 53%), blinding for participants or researchers was not conducted in nearly half (*k* = 8; 47%), and reporting of a manipulation check was unclear in over two-thirds (*k* = 13; 76%). Most studies reported randomization to treatment (k = 16; 94%), that all participants were included in the analysis (*k* = 16; 94%) and all studies adequately reported the results of all outcomes (*k* = 17; 100%). Risk of bias assessment is shown in Figure 2 and Supplementary material.

**Figure 2.**
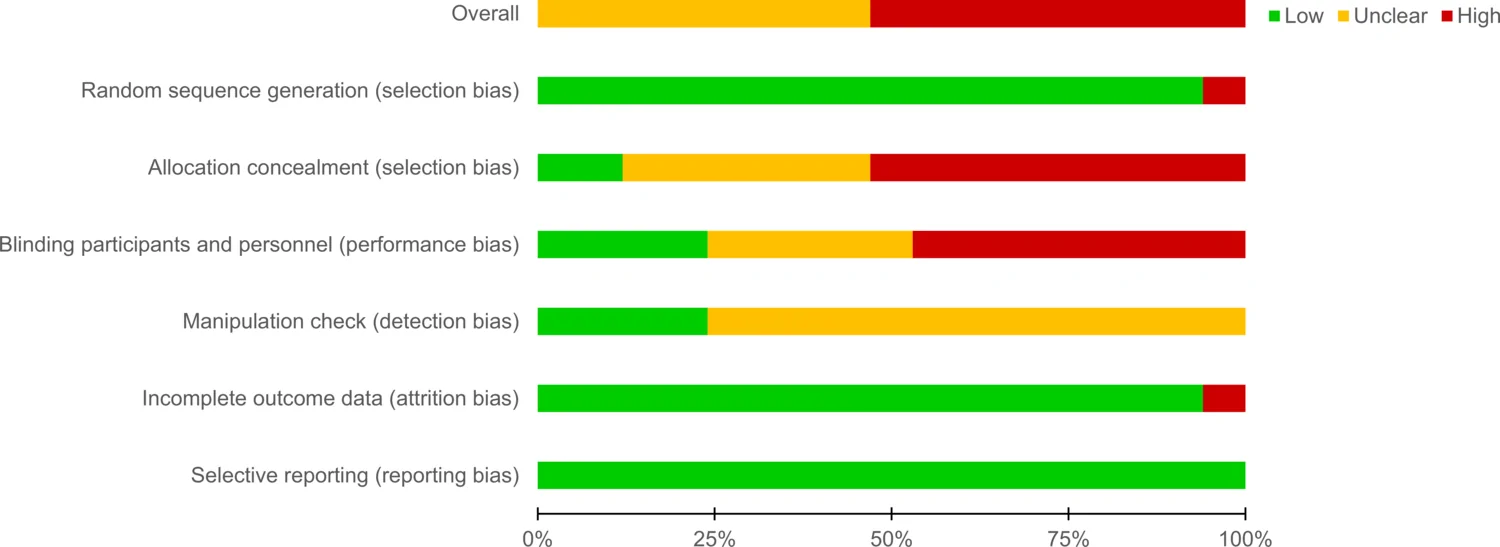
Risk of bias assessment.

### Results of synthesis

#### Primary outcomes

We conducted a meta-analysis of all primary outcomes reported in the 17 studies, which included 257 participants (Figure 3). Pooled estimates suggested a small-to-moderate effect of placebo caffeine benefiting sport performance (*d* = 0.49, 95% CI = 0.28 to 0.69, *p* < 0.001). There was a low degree of between-study heterogeneity (Q = 19.30, *p* = 0.21, *I* ^2^ = 21.03%) and no evidence of publication bias (Egger's test = 0.84 ± 1.31, *p* = 0.53). The FSN indicated that 107 studies were needed to reduce the pooled estimate to non-significance (Z = 5.28, *p* < 0.001) and sensitivity analysis revealed that the effect size did not change when one study was removed (*d* minimum to maximum = 0.43 to 0.53, *p* < 0.01).

**Figure 3.**
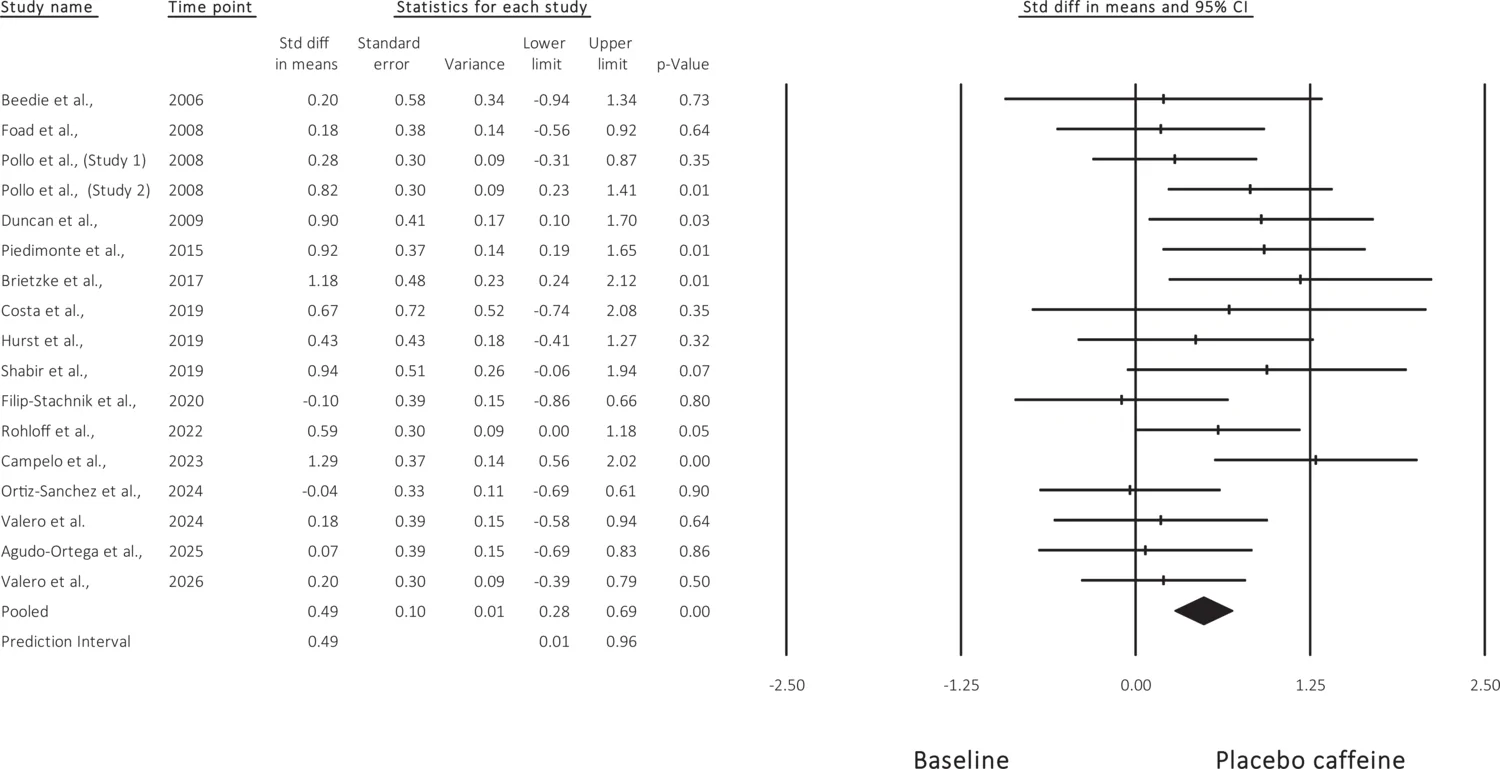
Pooled effect of placebo caffeine compared to baseline for primary outcomes of each included study.

Simple meta-regressions indicated that the pooled effect was likely to be moderated if researchers were blinded to treatment administration (b = 0.52 ± 0.24, 95% CI = 0.01 to 0.99, Z = 2.21, *p* = 0.03) and the proportion of females in the sample (b = −0.93 ± 0.38, 95% CI = −1.68 to −0.19, Z = −2.46, *p* = 0.01). That is, pooled effects were more likely to be larger when researchers were unaware that the substance was a placebo and in samples that had fewer females. No other variables were shown to moderate the pooled effect (see Supplementary material).

#### Sub-group analyses

Sufficient data were available to examine pooled effects on outcomes related to RPE, heart rate, power output, number of repetitions, velocity, total weight lifted, time-to-exhaustion, lactate, and oxygen consumption. An overview of all pooled effects for each secondary outcome analysis is shown in Table 2. Briefly, a significant large, pooled effect was reported for time-to-exhaustion, and moderate effects were found for RPE, number of repetitions and total weight lifted. No other significant pooled effects were reported.

**Table 2.** Subgroup meta-analyses for secondary outcomes.

Outcome	*k* (*n*)	*d* ± std error	Lower 95% CI	Upper 95% CI	*p*
RPE	11 (167)	0.52 ± 0.13	0.26	0.78	<0.01
Heart rate	6 (64)	0.21 ± 0.18	−0.13	0.55	0.23
Number of repetitions	5 (84)	0.48 ± 0.05	0.06	0.90	0.03
Power output	5 (60)	0.05 ± 0.18	−0.30	0.41	0.77
Velocity	4 (60)	−0.15 ± 0.18	−0.51	0.20	0.51
Time to complete	4 (54)	0.38 ± 0.19	0.00	0.75	0.05
Total weight lifted	3 (56)	0.73 ± 0.27	0.21	1.25	0.01
Oxygen consumption	3 (30)	0.51 ± 0.33	−0.14	1.16	0.13
Lactate	3 (29)	0.23 ± 0.27	−0.30	0.75	0.40
Time to exhaustion	2 (17)	1.07 ± 0.25	0.38	1.75	<0.01

Note: RPE = rating of perceived exertion, *k* = number of studies, *n* = sample size, *d* = Cohen's *d*, std error = standard error, CI = confidence interval.

For RPE, between-study heterogeneity (Q = 11.784, *p* = 0.30, *I* ^2^ = 15.14%) and evidence of publication bias (Egger's test = 2.56 ± 2.90, *p* = 0.40) was low, and the FSN indicated that 44 studies were needed to reduce the pooled estimate to non-significant (Z = 4.36 *p* < 0.001). Sensitivity analysis revealed that the effect size did not change when one study was removed (*d* minimum to maximum = 0.46 to 0.57, *p* < 0.001). Meta-regression revealed that number of manipulations to deceive participants about placebo caffeine moderated pooled effects (b = 0.21 ± 0.10, 95% CI = 0.01 to 0.41, Z = −2.09, *p* = 0.04), suggesting that more manipulations increased the pooled effect. Similarly, the mode of exercise moderated the effect when running was compared with weightlifting (b = −0.90 ± 0.38, 95% CI = −1.64 to −0.15, Z = −2.34, *p* = 0.02) but not cycling (b = −0.97 ± 0.57, 95% CI = −2.10 to 0.16, Z = −1.69, *p* = 0.09). This highlights that placebo effects on RPE may be higher during running than weightlifting tasks. Due to the low number of studies in the other sub-group analyses (*k* < 10), no other sensitivity analysis or meta-regressions were performed.

#### Certainty of evidence

As per the GRADE assessment, we assessed the imprecision, inconsistency, risk of publication bias, indirectness and risk of bias of the overall quality of evidence presented here. For our primary outcome analysis, results were consistent between trials, with studies sampling healthy adults who were, overall, physically active and there was low heterogeneity and risk of publication bias. However, there was high risk of bias across all studies, with no study being rated as low risk of bias, and some variation in the confidence interval. As such, it is likely that the overall quality of evidence falls within the low category, whereby the estimate of the effect is uncertain and future research using stronger research designs are needed. A summary of all GRADE assessments for each analysis is shown in Supplementary material, which ranged between low and very low certainty of evidence.

## Discussion

We found a small-to-moderate effect on sport performance when participants were deceptively administered a placebo and informed it is caffeine. These effects may be more likely to occur when researchers are blinded to the administration and in samples that recruit more male than female participants. We also found a large effect of placebo caffeine on time-to-exhaustion tasks, and moderate effects on RPE, the number of repetitions to lift a weight and the total amount of weight lifted. Low heterogeneity was reported for analyses and results are likely to be unaffected by publication bias. However, no study was rated as low risk of bias and as such, the certainty of evidence was graded low, suggesting that stronger research designs are required to provide greater confidence for the inferences reported here.

Like previous systematic reviews [3,5,13], placebo effects can improve sport performance when athletes receive a placebo and informed it is caffeine. Although caution should be made in comparison to other reviews of caffeine when differences in research design (e.g. randomized controlled trials vs. deceptive administration), and low certainty are evident, our pooled estimate (*d* = 0.49) is similar, and in some cases larger, than previous meta-analyses examining the administration of actual caffeine on exercise outcomes [18]. In their umbrella review of 21 meta-analyses, Grgic, Grgic [18] reported that effect sizes for caffeine on sport performance ranged from 0.16 to 0.63. This suggests that when an athlete receives caffeine and believes it is caffeine, the effectiveness of it could be attributed, in part, by placebo effects.

Our pooled effect estimate was moderated when researchers were blinded to the administration of the treatment. That is, effect sizes were larger when researchers were unaware that the treatment was a placebo. While this may seem counterintuitive (blinding researchers to an intervention is often used to reduce the occurrence of placebo effects [75–77]), it could be speculated that when a researcher administers a placebo and is aware it is a placebo, they may expect that it should not influence performance. This expectation could in turn, influence participant outcomes via (unconscious) changes in their behavior as a result of the researcher changing their instructions, body language and mannerisms during the trial [77]. When a researcher is unaware of what treatment is being administered, these changes in behavior may not occur and as such, they do not influence participant expectations. Although some have encouraged researchers to examine participant expectations [2,12,78], our data highlight the importance of examining researcher expectations about the treatment on reported outcomes. Similarly, while high risk of performance bias is suggested to be evident when participants are aware of the intervention they received [79], in placebo effect research this is potentially undesirable. That is, to heighten expectations, placebo effect researchers intentionally inform participants that they are to receive caffeine. Thus, blinding participants is likely to have been impractical for most studies included in this review and a reason for the high-risk of performance bias (Figure 2).

The proportion of females in the sample moderated pooled effects, whereby the fewer females recruited to a study, the greater the effect. Although this could indicate that placebo effects may be more likely to occur in males than females, our data is limited by both the high risk of bias found across studies and the lack of females represented in our analyses, which is typical in the dietary supplement literature [80]. From the 17 studies included in our review, only five sampled females, and from all data combined, 82% of participants were male. As such, there is a male bias in both the total number of participants and the number of single-sex studies examining placebo effects of caffeine on sport performance. The prevalence of males is higher than that reported in previous systematic reviews of placebo effects, which has been estimated at 69% [3], and in broader sport science and medicine research, whereby in journals published between 2014 and 2020, 66% of participants were male [81]. While there is evidence to highlight that males may be more likely to respond to placebo effects than females [82], we, like others [81,83] urge the sport science community to include more females in research so that we can better understand the impact placebo effects can have on both sexes.

Sub-group analyses revealed a moderate pooled effect on RPE. Evidence of placebo effects on exercise [84], pain [85], and cognition [86] highlights that improvements are often underpinned by subjective, self-appraised symptoms. Perceived exertion is a complex interaction of perceptual, cognitive and metabolic processes that moderate a number of psychosocial factors, including previous experiences, knowledge of the task, and memory [87]. In relation to placebo effects of caffeine, when a participant receives a placebo and informed it is caffeine, this may interact with psychosocial factors and increase participant expectation that they can, for example, run faster. These changes may be mediated by both neurobiological and physiological pathways [1], of which caffeine may also act [88]. Meta-regression analyses revealed that the pooled effect of RPE was moderated by the number of manipulations researchers used to influence participant expectations about the placebo (e.g. being told caffeine will improve performance, being provided with scientific evidence of the effectiveness of caffeine and a preconditioning procedure). As placebos are not administered in a vacuum [1], but in a complex setting consisting of several psychosocial stimuli, researchers can capitalize on various aspects of this setting to further augment participant expectations and in turn influence RPE.

### Limitations

A number of limitations associated with the evidence included in our review should be considered when interpreting our findings. First, high risk of bias was evident in over half of the studies. Allocation concealment was poor across most studies, whereby participants or researchers were aware of what treatment was to be received and when. Inadequate allocation concealment has been shown to influence treatment effect sizes [89]. Future research should ensure that when researchers are allocating participants to treatment conditions, they are blinded to this until after they have been recruited to the study. Similarly, nearly half of studies did not blind researchers to the treatment received. As we have shown here, when researchers are aware of what treatment participants received, this reduced the overall effect and suggest that researchers may (unconsciously) bias outcomes. It is therefore important for future research to ensure that when administering treatments to participants, researchers are blinded.

The evidence of this review is limited by the lack of female participants included across studies. While females are often excluded from repeated investigations in physical performance research due to variability in reproductive hormonal profiles [81], to provide better understanding of placebo caffeine on sport performance, a need exists for researchers to include more females in their samples. Our review is also limited by the fact that we only included studies written in English and peer-reviewed publications. There could be further studies in other languages or in grey literature (e.g. dissertation theses and conference abstracts), that we have not included, which could magnify the pooled effect [90,91]. As such, future research should expand the search strategy to studies written in languages other than English and peer-reviewed publications. Finally, six studies administered placebo caffeine less than 20 min prior to the task. Given that peak concentration of caffeine occurs between 30–60 min [15], some participants, who were aware of this, may have questioned the validity of the intervention and in turn, not believed that it would benefit performance. It is important that future placebo effect investigations administer placebo caffeine in line with the expected pharmacokinetic profile.

### Implications

Our data highlight that placebo effects could influence sport performance when athletes receive a placebo and believe it is caffeine. This emphasizes the importance of measuring and controlling for participant expectations about what has been received in both the analysis and interpretation of research findings. Several perceptual cues can indicate whether caffeine has been received (e.g. increase in heart rate, mental alertness). If a participant is aware prior to, or during the trial that they have received caffeine, this may influence their performance due to the expectation that caffeine was received [12]. Equally, if a participant is aware that they have received a placebo, this could negatively influence observed outcomes.

From a practical perspective our data highlight the importance of engendering a positive expectation about the effectiveness of caffeine during the administration to athletes. As it is unethical to deceptively administer a placebo in practice [92,93], it is important that during the administration of actual caffeine, the athlete fully expects it to improve their performance. Placebo effects often interact with the verum effects of caffeine [94], and as such it is important that the placebo effect component of caffeine is capitalized on to ensure that the benefits are maximized.

## Supplementary Material

Supplementary Material Supplementary_materials.zip

## Data Availability

All data is available in the supplementary material.

## Endnotes

^1^
For brevity, we use the term “placebo caffeine” to indicate placebo described as caffeine.

^2^
All studies used a baseline condition. As such, we did not calculate the difference between placebo caffeine no-treatment control.

^3^
Pollo et al. (2008) was a multi-study project involving two different experimental samples and treated as two separate studies.

## Registration and protocol

This systematic review and meta-analysis was published and registered to the Open Science Framework (OSF) registries (doi: 10.17605/OSF.IO/W8KY6). Minor changes were made following the publication of the protocol. First, two authors were identified to select the primary and secondary outcomes, however, this changed to three. Second, as most studies were cross-over trials (13 of the 17), SMDs were computed between baseline and placebo caffeine trials instead of between placebo caffeine and control.
