# Supplementary material for: Placebo effects of caffeine on sport performance: a systematic review and meta-analysis
Source: J Int Soc Sports Nutr. 2026 Oct 20;23(1):2747142. doi: 10.1080/15502783.2026.2747142 (PMC13686187; doi:10.1080/15502783.2026.2747142)
Supplement: Supplementary file 1 [file RSSN_A_2747142_SM2207.zip › 2 Search strategy.docx]

**Search Strategy**

| **Database searched** | **Platform** | **Years of coverage** | **Records** | **Records after duplicates removed** |
| --- | --- | --- | --- | --- |
| Medline ALL | Ovid | 1946 - Present | 632 | 624 |
| Embase | Embase.com | 1971 - Present | 948 | 429 |
| Web of Science Core Collection* | Web of Knowledge | 1900 - Present | 951 | 415 |
| CINAHL Plus | EBSCO | 1982 - Present | 268 | 39 |
| **Total** | | | **2799** | **1507** |

*Science Citation Index Expanded (SCI-EXPANDED) 1900-present ; Social Sciences Citation Index (SSCI) 1900-present ; Arts & Humanities Citation Index (AHCI) 1975-present ; Conference Proceedings Citation Index – Science (CPCI-S) 1990-present ; Conference Proceedings Citation Index – Social Science & Humanities (CPCI-SSH) 1990-present ; Book Citation Index – Science (BKCI-S) 2005-present ; Book Citation Index – Social Sciences & Humanities (BKCI-SSH) 2005-present ; Emerging Sources Citation Index (ESCI) 2005-present ; Current Chemical Reactions (CCR-EXPANDED) 1985-present ; Index Chemicus (IC) 1993-present
No other database limits were used than those specified in the search strategies

**medline**

(exp Sports/ OR exp Athletes/ OR Athletic Performance/ OR Physical Functional Performance/ OR exp Exercise Test/ OR exp Exercise/ OR Physical Fitness / OR exp Psychomotor Performance/ OR exp Motor Activity/ OR exp Muscle Strength/ OR Fatigue/ OR Physical Endurance / OR (sport* OR athlet* OR exertion* OR exercis* OR fitness OR cycling OR running OR strength OR fatigue* OR ((endurance* OR physical* OR jump) ADJ3 performan*) OR ((psychomotor* OR motor* OR physical) ADJ3 activit*) OR performan* OR Athletic* OR baseball* OR basketball* OR boating* OR body-build* OR bowling* OR boxing* OR calisthenic* OR car-racing* OR climbing* OR cricket* OR cross-training* OR cycling* OR diving* OR endurance* OR fencing* OR football* OR golf* OR gymnastic* OR hockey* OR horseback-riding* OR ice-hockey* OR jogging* OR kickboxing* OR lacrosse* OR martial-art* OR motocross* OR motorcycle-racing* OR mountaineering* OR nordic-walking* OR racewalking* OR racquet* OR rock-climbing* OR roller-skating* OR rugb* OR running* OR skateboarding* OR skating* OR skiing* OR soccer* OR softball* OR squash* OR surfing* OR swimming* OR tennis* OR triathlon* OR volleyball* OR water-aerobic* OR water-polo* OR waterpolo* OR water-skiing* OR waterskiing* OR watersport* OR weight-lifting* OR weightlifting* OR wintersport* OR wrestling* OR yoga* OR aikido* OR jiu-jitsu* OR judo* OR karate* OR kung-fu* OR qigong* OR taekwondo* OR Tai-Chi* OR TaiChi* OR Tai-Ji* OR Taiji* OR wushu*).ab,ti,kw.) AND (Placebo Effect/ OR * Placebos/ OR * Perception/ OR * Motivation/ OR (placebo*-effect* OR nocebo*-effect* OR sham OR deceit* OR decepti* OR fake OR false OR overestimat* OR over-estimat* OR exaggerat* OR ((belief* OR believe* OR expectation* OR expectanc* OR perceive* OR perception* OR motivation* OR suggested* OR primed OR priming OR told OR manipul*) ADJ3 caffein*)).ab,ti,kw. OR (placebo* OR belief* OR believe* OR expectation* OR expectanc* OR perceive* OR perception* OR motivation* OR suggest* OR primed OR priming OR told OR manipul*).ti.) AND (Caffeine / OR Energy Drinks / OR Coffee / OR (caffein* OR energy-drink* OR exercise-drink* OR sport-drink* OR sports-drink* OR red-bull OR coffee* OR bang-energy OR Monster).ab,ti,kw.) NOT (exp animals/ NOT humans/)

**embase**

(sport/exp OR athlete/exp OR 'athletic performance'/de OR 'physical performance'/de OR 'exercise test'/exp OR exercise/exp OR fitness/de OR 'motor performance'/de OR 'psychomotor activity'/exp OR 'physical activity'/de OR strength/exp OR fatigue/de OR endurance/de OR (sport* OR athlet* OR exertion* OR exercis* OR fitness OR cycling OR running OR strength OR fatigue* OR ((endurance* OR physical* OR jump) NEAR/3 performan*) OR ((psychomotor* OR motor* OR physical) NEAR/3 activit*) OR performan* OR Athletic* OR baseball* OR basketball* OR boating* OR body-build* OR bowling* OR boxing* OR calisthenic* OR car-racing* OR climbing* OR cricket* OR cross-training* OR cycling* OR diving* OR endurance* OR fencing* OR football* OR golf* OR gymnastic* OR hockey* OR horseback-riding* OR ice-hockey* OR jogging* OR kickboxing* OR lacrosse* OR martial-art* OR motocross* OR motorcycle-racing* OR mountaineering* OR nordic-walking* OR racewalking* OR racquet* OR rock-climbing* OR roller-skating* OR rugb* OR running* OR skateboarding* OR skating* OR skiing* OR soccer* OR softball* OR squash* OR surfing* OR swimming* OR tennis* OR triathlon* OR volleyball* OR water-aerobic* OR water-polo* OR waterpolo* OR water-skiing* OR waterskiing* OR watersport* OR weight-lifting* OR weightlifting* OR wintersport* OR wrestling* OR yoga* OR aikido* OR jiu-jitsu* OR judo* OR karate* OR kung-fu* OR qigong* OR taekwondo* OR Tai-Chi* OR TaiChi* OR Tai-Ji* OR Taiji* OR wushu*):ab,ti,kw) AND ('placebo effect'/de OR 'sham procedure'/de OR placebo/mj OR perception/mj OR expectancy/mj OR motivation/mj OR (placebo*-effect* OR nocebo*-effect* OR sham OR deceit* OR decepti* OR fake OR false OR overestimat* OR over-estimat* OR exaggerat* OR ((belief* OR believe* OR expectation* OR expectanc* OR perceive* OR perception* OR motivation* OR suggested* OR primed OR priming OR told OR manipul*) NEAR/3 caffein*)):ab,ti,kw OR (placebo* OR belief* OR believe* OR expectation* OR expectanc* OR perceive* OR perception* OR motivation* OR suggest* OR primed OR priming OR told OR manipul*):ti) AND (caffeine/de OR 'energy drink'/de OR coffee/exp OR 'sports drink'/de OR 'caffeine intake'/de OR (caffein* OR energy-drink* OR exercise-drink* OR sport-drink* OR sports-drink* OR red-bull OR coffee* OR bang-energy OR Monster):ab,ti,kw) NOT (([Conference Abstract]/lim OR [Conference Review]/lim OR [preprint]/lim OR 'clinical trial':it) AND [1800-2023]/py) NOT ([animals]/lim NOT [humans]/lim)

**Web of science**

TS=((sport* OR athlet* OR exertion* OR exercis* OR fitness OR cycling OR running OR strength OR fatigue* OR ((endurance* OR physical* OR jump) NEAR/2 performan*) OR ((psychomotor* OR motor* OR physical) NEAR/2 activit*) OR performan* OR Athletic* OR baseball* OR basketball* OR boating* OR body-build* OR bowling* OR boxing* OR calisthenic* OR car-racing* OR climbing* OR cricket* OR cross-training* OR cycling* OR diving* OR endurance* OR fencing* OR football* OR golf* OR gymnastic* OR hockey* OR horseback-riding* OR ice-hockey* OR jogging* OR kickboxing* OR lacrosse* OR martial-art* OR motocross* OR motorcycle-racing* OR mountaineering* OR nordic-walking* OR racewalking* OR racquet* OR rock-climbing* OR roller-skating* OR rugb* OR running* OR skateboarding* OR skating* OR skiing* OR soccer* OR softball* OR squash* OR surfing* OR swimming* OR tennis* OR triathlon* OR volleyball* OR water-aerobic* OR water-polo* OR waterpolo* OR water-skiing* OR waterskiing* OR watersport* OR weight-lifting* OR weightlifting* OR wintersport* OR wrestling* OR yoga* OR aikido* OR jiu-jitsu* OR judo* OR karate* OR kung-fu* OR qigong* OR taekwondo* OR Tai-Chi* OR TaiChi* OR Tai-Ji* OR Taiji* OR wushu*)) AND (TS=(placebo*-effect* OR nocebo*-effect* OR sham OR deceit* OR decepti* OR fake OR false OR overestimat* OR over-estimat* OR exaggerat* OR ((belief* OR believe* OR expectation* OR expectanc* OR perceive* OR perception* OR motivation* OR suggested* OR primed OR priming OR told OR manipul*) NEAR/2 caffein*)) OR TI=(placebo* OR belief* OR believe* OR expectation* OR expectanc* OR perceive* OR perception* OR motivation* OR suggest* OR primed OR priming OR told OR manipul*)) AND TS=((caffein* OR energy-drink* OR exercise-drink* OR sport-drink* OR sports-drink* OR red-bull OR coffee* OR bang-energy OR Monster)) AND DT=(article)

**Cinahl**

(MH Sports+ OR MH Athletes+ OR MH Athletic Performance OR MH Physical Performance OR MH Exercise Test+ OR MH Exercise+ OR MH Physical Fitness OR MH Psychomotor Performance+ OR MH Motor Activity+ OR MH Muscle Strength+ OR MH Fatigue OR MH Physical Endurance OR TI(sport* OR athlet* OR exertion* OR exercis* OR fitness OR cycling OR running OR strength OR fatigue* OR ((endurance* OR physical* OR jump) N2 performan*) OR ((psychomotor* OR motor* OR physical) N2 activit*) OR performan* OR Athletic* OR baseball* OR basketball* OR boating* OR body-build* OR bowling* OR boxing* OR calisthenic* OR car-racing* OR climbing* OR cricket* OR cross-training* OR cycling* OR diving* OR endurance* OR fencing* OR football* OR golf* OR gymnastic* OR hockey* OR horseback-riding* OR ice-hockey* OR jogging* OR kickboxing* OR lacrosse* OR martial-art* OR motocross* OR motorcycle-racing* OR mountaineering* OR nordic-walking* OR racewalking* OR racquet* OR rock-climbing* OR roller-skating* OR rugb* OR running* OR skateboarding* OR skating* OR skiing* OR soccer* OR softball* OR squash* OR surfing* OR swimming* OR tennis* OR triathlon* OR volleyball* OR water-aerobic* OR water-polo* OR waterpolo* OR water-skiing* OR waterskiing* OR watersport* OR weight-lifting* OR weightlifting* OR wintersport* OR wrestling* OR yoga* OR aikido* OR jiu-jitsu* OR judo* OR karate* OR kung-fu* OR qigong* OR taekwondo* OR Tai-Chi* OR TaiChi* OR Tai-Ji* OR Taiji* OR wushu*) OR AB(sport* OR athlet* OR exertion* OR exercis* OR fitness OR cycling OR running OR strength OR fatigue* OR ((endurance* OR physical* OR jump) N2 performan*) OR ((psychomotor* OR motor* OR physical) N2 activit*) OR performan* OR Athletic* OR baseball* OR basketball* OR boating* OR body-build* OR bowling* OR boxing* OR calisthenic* OR car-racing* OR climbing* OR cricket* OR cross-training* OR cycling* OR diving* OR endurance* OR fencing* OR football* OR golf* OR gymnastic* OR hockey* OR horseback-riding* OR ice-hockey* OR jogging* OR kickboxing* OR lacrosse* OR martial-art* OR motocross* OR motorcycle-racing* OR mountaineering* OR nordic-walking* OR racewalking* OR racquet* OR rock-climbing* OR roller-skating* OR rugb* OR running* OR skateboarding* OR skating* OR skiing* OR soccer* OR softball* OR squash* OR surfing* OR swimming* OR tennis* OR triathlon* OR volleyball* OR water-aerobic* OR water-polo* OR waterpolo* OR water-skiing* OR waterskiing* OR watersport* OR weight-lifting* OR weightlifting* OR wintersport* OR wrestling* OR yoga* OR aikido* OR jiu-jitsu* OR judo* OR karate* OR kung-fu* OR qigong* OR taekwondo* OR Tai-Chi* OR TaiChi* OR Tai-Ji* OR Taiji* OR wushu*)) AND (MH Placebo Effect OR MM Placebos OR MM Perception OR MM Motivation OR AB(placebo*-effect* OR nocebo*-effect* OR sham OR deceit* OR decepti* OR fake OR false OR overestimat* OR over-estimat* OR exaggerat* OR ((belief* OR believe* OR expectation* OR expectanc* OR perceive* OR perception* OR motivation* OR suggested* OR primed OR priming OR told OR manipul*) N2 caffein*)) OR TI(placebo* OR belief* OR believe* OR expectation* OR expectanc* OR perceive* OR perception* OR motivation* OR suggest* OR primed OR priming OR told OR manipul*)) AND (MH Caffeine OR MH Energy Drinks + OR MH Coffee OR TI(caffein* OR energy-drink* OR exercise-drink* OR sport-drink* OR sports-drink* OR red-bull OR coffee* OR bang-energy OR Monster) OR AB(caffein* OR energy-drink* OR exercise-drink* OR sport-drink* OR sports-drink* OR red-bull OR coffee* OR bang-energy OR Monster)) NOT (MH animals+ NOT MH humans+)
