# Supplementary material for: Placebo effects of caffeine on sport performance: a systematic review and meta-analysis
Source: J Int Soc Sports Nutr. 2026 Oct 20;23(1):2747142. doi: 10.1080/15502783.2026.2747142 (PMC13686187; doi:10.1080/15502783.2026.2747142)
Supplement: Supplementary file 1 [file RSSN_A_2747142_SM2207.zip › 4 Results of individual studies.docx]

**Results of individual studies**

Beedie et al. (2006) recruited trained cyclists (N = 6) to complete 10-km cycling time-trials. Participants completed baseline in the first and final trial, whereas in in the other three trials, participants received a placebo and were informed it could be: 1) placebo, 2) 4.5 mg·kg-1of caffeine and 3) 9.0 mg·kg-1of caffeine. Prior to completing trials, authors provided participants with scientific literature of caffeine on cycling performance and described anecdotal evidence of the use of caffeine amongst elite cyclists. Although not statistically significant, participants’ mean power output increased by 1.3% and 3.1% when they believed they had ingested 4.5 and 9.0 mg·kg-1 of caffeine, respectively. While no differences were reported between any of the placebo trials and baseline in blood-lactate, mean heart rate or mean oxygen consumption, semi-structured interviews indicated that changes in performance were related to an increase in expectations, pain reduction, greater attention and increased arousal.

Foad et al. (2008) used a cross-over, balanced placebo design to examine placebo effects of 5.0 mg·kg-1 caffeine during 40-km cycling time-trials. Using this design, authors assessed each possible combination of what a participant believed they had taken and what they had actually taken across four conditions: 1) told caffeine/received caffeine, 2) told caffeine/received placebo, 3) told placebo/given caffeine and 4) told placebo/given placebo. Prior to completing any trials, participants (N = 14) attended an in-person, 90-minute presentation about the benefits of caffeine, which included an overview of scientific research into caffeine and cycling performance, as well as anecdotal evidence relating to the use of caffeine amongst elite cyclists. Compared to baseline trials, authors reported no changes in mean power output when participants received a placebo and informed it was caffeine (p>0.05), whereas when they received caffeine, power output improved when they were informed it was caffeine (p<0.05) and when it was a placebo (p<0.01). No differences (p>0.05) were reported in any conditions for mean oxygen uptake, heart rate and blood lactate.

Across two studies, Pollo et al. (2008) examined the placebo effect of caffeine during a leg extension task at 60% 1-rep max (1RM). In Study 1, participants completed baseline trials of total work performance (kj), number of repetitions (n) and rating of perceived exertion (RPE), before being randomised to a placebo (n = 11) or control (n = 11) condition. In the placebo condition, participants were provided with scientific papers about the benefits of caffeine and verbally informed that it would improve performance. They then received decaffeinated coffee and informed it was a high dose of caffeine. Control condition participants received no treatment or information about caffeine. Participants then completed experimental trials in the same manner as baseline. Results showed that in the placebo group, total work performed, and number of repetitions significantly improved compared to baseline (p=0.01) and controls (p=0.04), whereas no differences were reported in RPE (p>0.05). In Study 2, authors used the same leg extension task and baseline trial as Study 1 before randomising participants to a preconditioning group (n = 11) or control (n = 11). In the preconditioning group, participants received the same placebo caffeine and information about caffeine’s benefits in Study 1. They then performed experimental trials whereby the weight lifted was surreptitiously reduced to 45% 1RM in a second and third trial and returned to 60% 1RM in a final trial. Participants in the control group did not receive a placebo and performed a further three baseline trials. In the preconditioning condition, the weight lifted, and number of repetitions performed was significantly higher than baseline (p=0.002) when the load was restored to 60% 1RM. No differences were reported between controls (p=0.074). Significant differences were reported in RPE between baseline and experimental trials in the preconditioning condition (p=0.01).

Using a similar measure as Pollo et al. (2008), Duncan et al. (2009) told participants (N = 12) that they were to receive, in a randomised order, 3.0 mg·kg-1 of caffeine and a placebo before completing a 60% 1RM leg extension task to failure. However, in both conditions, participants were given a placebo. Participants received scientific papers about the benefit of caffeine and suggestions that it would improve their performance. In comparison to baseline, results showed that when participants believed they had ingested caffeine, the number of repetitions and total weight lifted improved significantly (p<0.05). These changes were not shown when participants believed they had received a placebo. While authors measured systolic and diastolic blood pressure, peak heart rate and RPE, these were not significant compared to baseline (p>0.05).

Piedimonte et al. (2015) examined whether placebo effects of caffeine can affect the preparatory phase of movement and readiness potential during a 30-minute, finger flexion task at 60% 1RM. Authors used a parallel design and randomly assigned participants to two conditions: 1) placebo (n = 15) or 2) control (n = 15). Participants in the placebo condition completed 10-minutes of the finger flexion task, before being administered 20ml of decaffeinated coffee that that was informed to be a high dose of caffeine, which would benefit their performance in the final 10-minutes of the task. Participants then completed the rest of the task and authors measured RPE and readiness potential of movement using electroencephalogram (EEG). Control participants were not administered a placebo and told to complete the task for 30-minutes. Results showed that during the last 10-minutes of the task in the placebo condition, RPE reduced significantly compared to the previous 10-minutes (p<0.001), whereas in controls it significantly increased (p<0.001). The changes in RPE were moderated by readiness potential, whereby amplitudes were significantly greater in controls but remained unchanged in the placebo condition.

Brietzke et al. (2017) examined the placebo effect of caffeine during a cycling maximal incremental test. After completing familiarisation, participants (N = 9) completed three conditions in a randomised order: 1) baseline, 2) caffeine or 3) placebo. In the placebo condition, participants received a placebo and were led to believe that they were ingesting 6.0 mg·kg-1 caffeine that has the potential to improve their performance. In comparison to baseline, time-to-exhaustion and peak power output significantly improved in both the caffeine (p<0.05) and placebo (p<0.05) conditions. No changes in VO2max or RPE were reported between any of the conditions (p>0.05).

In a randomised, double-blind study involving Paralympic weightlifters (N = 4), Costa et al. (2019) assessed placebo effects of caffeine during submaximal (50-, 60-, 70-, 80% 1RM) bench press. After establishing 1RM, in a randomised order, participants completed submaximal trials with either the ingestion of a placebo capsule believed to be 6.0 mg·kg-1 of caffeine or nothing (i.e., baseline). Compared to baseline, no improvements (p>0.05) were shown at 60, 70 or 80% 1RM in any measure (i.e., mean velocity, mean velocity to peak, mean propulsive velocity or peak velocity), whereas, changes were shown at 50% 1RM for mean velocity (effect size = 0.36, p<0.05) and mean propulsive velocity (effect size = 0.46, p<0.05).

Using the balanced placebo design, Hurst, Schiphof-Godart, Hettinga, et al. (2020) examined the placebo effect of 3.0mg·kg-1of caffeine during 1,000m running time-trials. Prior to data collection, participants (N = 11) were given a short presentation on the benefits of caffeine, provided with scientific and anecdotal evidence of its ergogenic effects and informed that it used to be a prohibited performance enhancing substance. They then completed five (1. Baseline, 2. told caffeine/given caffeine, 3. told caffeine/given placebo, 4. told placebo/given caffeine, 5. told placebo/given placebo) 1000-m time trials as fast as possible. Compared to baseline, when participants believed they had ingested caffeine but administered a placebo, time to complete the 1000-m distance significantly improved (p<0.01). This change was similar to when participants received caffeine and believed it was caffeine (p>0.05). Authors reported that improvements in performance were related to faster times in the first 400-m of the distance. No changes in peak heart rate or RPE were reported between conditions.

Shabir et al. (2019) used the balanced placebo design to examined placebo effects of 3.0mg·kg-1 of caffeine during stimulated soccer performance. Participants (N = 8) were provided with a manuscript and watched a video about the benefits of caffeine, before completing a number of baseline trials including: the Loughborough Intermittent Shuttle test, Loughborough soccer passing test and a dynamic reaction time test. They then completed all trials again under the four balanced placebo design conditions (i.e., told caffeine/given caffeine, told caffeine/given placebo, told placebo/given caffeine, told placebo/given placebo) in a randomised order. Authors reported that when participants received a placebo but informed caffeine, compared to baseline, time-to-exhaustion and heart rate was significantly lower during the Loughborough Intermittent Shuttle test (p>0.05). No changes were reported in lactate or RPE, as well dynamic reaction time or in the Loughborough Soccer Passing Test (p>0.05). Authors also administered psychometrics related to caffeine expectations and profile of mood states but reported no differences between any conditions (p>0.05).

Filip-Stachnik et al. (2020) recruited resistance trained females (N = 13) and examined placebo effects of caffeine on 1RM and 50% 1RM bench press. Participants were verbally informed by a qualified dietician that they were to receive 6.0 mg·kg-1 of caffeine, which would benefit their performance, and given scientific evidence about caffeine’s ergogenic effect. In comparison to baseline, no change in 1RM was reported (p = 0.10). Similar results were shown during the 50% 1RM test for the number of repetitions, time under tension, mean and peak power output, and mean and peak velocity (all p>0.05).

Rohloff et al. (2020) used a double-blind, randomized cross-over design to determine the effectiveness of a placebo described as caffeine on 4-km time-performance. Twenty-two healthy male runners completed baseline and two experimental conditions. In experimental conditions, participants completed the 4-km time-trial with a placebo described as 4.0 mg·kg^-1^ of caffeine and 4.0 mg·kg^-1^ of caffeine. Results showed that compared to baseline, time to complete the 4-km time-trial was faster when participants received a placebo. No differences between placebo and caffeine were shown, suggesting that the benefits of caffeine on time-trial performance may be related to the belief it has been received. Participants in the placebo conditions also ran faster in the 1^st^ km of the trial compared to baseline and caffeine. No differences in RPE were reported between any conditions.

Campelo et al. (2023) examined whether the number of repetitions and RPE during 80% 1RM bench press would change if participants (N = 15) received a placebo believed to be caffeine or lactic acid. Authors provided education about the benefits of caffeine and the harmful effects of lactic acid via a short video presentation that they watched on their mobile phones. Results showed that compared to baseline, a significant increase in the number of repetitions was reported when participants believed they had ingested caffeine (p<0.05). These improvements were associated with decreased in RPE (p<0.05). The opposite results were shown in the lactic acid condition, whereby participants reported decreases in the number of repetitions and increases in RPE compared to baseline (p<0.05).

After establishing participants (N = 18) 1RM for both bench press and back squat, Ortiz-Sanchez et al. (2024) compared a placebo compared to baseline during 3 repetitions of bench and back squat at 50-, 75- and 90% 1RM. In the placebo condition, participants were told that they were to receive a high dose of 9.0 mg·kg-1of caffeine and authors measured several variables including velocity, power output, force and heart rate variability. In comparison to baseline, only two significant effects were reported: 1) mean velocity of bench press at 50% 1RM and 2) the rate of force development of back squat at 75% 1RM. All other variables for each intensity were not significant (p>0.05). Authors also measured perceptions of performance and prevalence of side effects following ingestion of the placebo. Authors reported that while participants did not perceive their performance to be affected (e.g., power, endurance and exertion), they felt less nervous and more active, and self-reported an increase in heart rate and urine production.

Valero et al. (2024) administered placebo caffeine chewing gum to participants (N = 13) and compared it to baseline during a 6-minute maximal running test. Participants were informed that caffeine chewing gum can improve performance and were told to chew for 5-minutes prior to the trial. Compared to baseline, the distance covered was significantly greater in the placebo condition (p = 0.03), which was associated with an increase in speed at the 800- (p <0.03) and 1200-m (p = 0.04) split. Authors reported no significant differences for heart rate (p = 0.12), RPE (p = 0.39), kinematic variables (i.e., step frequency, vertical oscillation, foot contact time; p >0.05) or report side-effects (e.g., nervousness, muscular pain and headache) between conditions.

Agudo-Ortega et al (2025) investigated the placebo effect of caffeine on 60-m sprint and standing triple jump performance. Using a within-participant, counterbalanced design, authors gave capsules to participants and informed them it was caffeine. After completing a standardised warm up, participants completed a standing triple jump and then ran a 60-m sprint as fast as possible. Following the placebo condition, participants were asked to complete a caffeine-related side-effects questionnaire. Results showed that compared to baseline, no differences in any measures were reported (p >0.05). Authors mentioned that over half of participants reported feeling active after the placebo described as caffeine. Whereas 3 and 2 participants reported feeling nervous and insomnia, respectively.

Valero et al. (2026) administered placebos described as 3.0 mg·kg-1 of caffeine to physically active young adults. Using a crossover, counterbalanced design, participants completed a battery of measurements including number of repetitions for bench press at 75% 1RM, propulsive velocity of bench press at 25, 50 and 75% 1RM, peak countermovement jump, number of repeated jumps and side effects related to ingestion of caffeine. Compared to baseline, participants reported improvements in the number of bench press repetitions and mean velocity at 75% 1RM. No differences were found for mean velocity at 25 and 50% 1RM, or for peak countermovement jump height and number of repeated jumps. Authors reported that 8 participants reported feeling active and 3 reported nervous after taking the placebo.
