# Supplementary material for: Placebo effects of caffeine on sport performance: a systematic review and meta-analysis
Source: J Int Soc Sports Nutr. 2026 Oct 20;23(1):2747142. doi: 10.1080/15502783.2026.2747142 (PMC13686187; doi:10.1080/15502783.2026.2747142)
Supplement: Supplementary file 1 [file RSSN_A_2747142_SM2207.zip › 7 Moderator analyses.docx]

| **Supplementary table. Primary outcome moderator analyses** | |  |  |  |  |  |
| --- | --- | --- | --- | --- | --- | --- |
| **Moderator** | **Categorical Comparator** | **β** | **SE** | **95% CI** | ***Z*** | ***p*** |
| Type of exercise (Cycling) | Running | −0.12 | 0.38 | −0.62 to 0.86 | −0.32 | 0.75 |
|  | Weightlifting | −0.11 | 0.33 | −0.75 to 0.54 | −0.33 | 0.75 |
| Type of exercise (Running) | Weightlifting | −0.23 | 0.31 | −0.84 to 0.38 | −0.74 | 0.46 |
| Duration of exercise (Long) | Moderate | −0.40 | 0.32 | −1.03 to 0.22 | 1.27 | 0.21 |
|  | Short | −0.16 | 0.81 | −1.76 to 1.44 | −0.20 | 0.85 |
| Duration of exercise (Moderate) | Short | 0.33 | 0.74 | −1.23 to 1.78 | 0.44 | 0.66 |
| Sample ability (trained) | Untrained | −0.15 | 0.26 | −0.60 to 0.37 | −0.56 | 0.57 |
| Proportion of females | − | −0.81 | 0.39 | −1.59 to −0.01 | −2.06 | 0.04 |
| Mean age | − | 0.00 | 0.02 | −0.01 to 0.03 | −0.26 | 0.79 |
| Study design (Between participant) | Within participant | −0.11 | 0.27 | −0.65 to 0.42 | −0.42 | 0.68 |
| Researcher blinded (No) | Yes | 0.45 | 0.23 | 0.00 to 0.91 | 1.97 | 0.04 |
| Randomisation (No) | Yes | 0.00 | 0.35 | −0.69 to 0.69 | 0.01 | 0.99 |
| Familiarisation (No) | Yes | −0.30 | −0.26 | −0.82 to 0.21 | −1.15 | 0.25 |
| Control group (No) | Yes | −0.11 | 0.27 | −0.42 to 0.64 | 0.42 | 0.68 |
| Type of placebo (capsule) | Coffee | 0.22 | 0.27 | −0.32 to 0.74 | 0.99 | 0.43 |
|  | Saline | 0.34 | 0.34 | −0.33 to 1.00 | 0.99 | 0.32 |
| Type of placebo (Coffee) | Saline | 0.12 | 0.42 | −0.71 to 0.96 | 0.29 | 0.77 |
| Perceived dose | − | −0.03 | 0.05 | −0.12 to 0.06 | −0.67 | 0.51 |
| Timing of administration (<1 minute) | 1 to 20 minutes | 0.16 | 0.30 | −0.43 to 0.74 | 0.52 | 0.60 |
|  | >20 minutes | 0.38 | 0.54 | −1.44 to 0.68 | 0.70 | 0.48 |
| Timing of administration (1 to 20 minutes) | >20 minutes | −0.42 | 0.48 | −1.37 to 0.51 | −0.90 | 0.37 |
| Actual caffeine (No) | Yes | 0.22 | 0.24 | −0.24 to 0.68 | 0.94 | 0.35 |
| Number of manipulations | − | 0.03 | 0.10 | −0.15 to 0.22 | 0.34 | 0.73 |
| Risk of bias | − | −0.17 | 0.26 | −0.68 to 0.34 | 0.66 | 0.51 |

| **Supplementary table. Rating of perceived exertion sub-group moderator analyses** | | | | | | |
| --- | --- | --- | --- | --- | --- | --- |
| Moderator | Categorical Comparator | β | SE | 95% CI | *Z* | *p* |
| Type of exercise (Cycling) | Running | −0.96 | 0.57 | −2.09 to 0.16 | −1.69 | 0.09 |
|  | Weightlifting | 0.07 | 0.51 | −0.92 to 1.01 | 0.14 | 0.90 |
| Type of exercise (Running) | Weightlifting | 0.89 | 0.38 | −1.65 to −0.15 | −2.34 | 0.02 |
| Duration of exercise (Moderate) | Short | −0.30 | 0.46 | −1.97 to 0.60 | −0.66 | 0.51 |
| Sample ability (trained) | Untrained | −0.57 | 0.33 | −1.21 to 0.07 | −1.75 | 0.08 |
| Proportion of females | − | −1.60 | 1.32 | −4.19 to 1.00 | −1.21 | 0.23 |
| Mean age | − | 0.07 | 0.90 | −0.11 to 0.24 | 0.74 | 0.46 |
| Study design (Between participant) | Within participant | 0.10 | 0.45 | −0.78 to 0.98 | 0.22 | 0.83 |
| Researcher blinded (No) | Yes | 0.10 | 0.41 | −0.71 to 0.91 | 0.23 | 0.82 |
| Randomisation (No) | Yes | −0.94 | 0.54 | −1.99 to 0.11 | −0.18 | 0.08 |
| Familiarisation (No) | Yes | 0.25 | 0.56 | −0.85 to 1.34 | 0.44 | 0.66 |
| Control group (No) | Yes | 0.10 | 0.45 | −0.78 to 0.98 | 0.22 | 0.83 |
| Type of placebo (capsule) | Coffee | −0.02 | 0.54 | −1.07 to 1.03 | 0.04 | 0.97 |
|  | Saline | 0.23 | 0.58 | −0.90 to 1.37 | 0.40 | 0.66 |
| Type of placebo (Coffee) | Saline | 0.22 | 0.53 | −0.82 to 1.25 | 0.41 | 0.69 |
| Perceived dose | − | −0.10 | 0.06 | −0.21 to 0.02 | −1.55 | 0.12 |
| Timing of administration (<1 minute) | >20 minutes | 0.10 | 0.45 | −0.78 to 0.98 | 0.22 | 0.83 |
| Actual caffeine (No) | Yes | 0.58 | 0.33 | 0.07 to 1.21 | 1.74 | 0.08 |
| Number of manipulations | − | 0.21 | 0.10 | 0.01 to 0.41 | 2.09 | 0.04 |
| Risk of bias | − | 0.09 | 0.24 | −0.37 to 0.55 | 0.39 | 0.70 |
