# Supplementary material for: Placebo effects of caffeine on sport performance: a systematic review and meta-analysis
Source: J Int Soc Sports Nutr. 2026 Oct 20;23(1):2747142. doi: 10.1080/15502783.2026.2747142 (PMC13686187; doi:10.1080/15502783.2026.2747142)
Supplement: Supplementary file 1 [file RSSN_A_2747142_SM2207.zip › 8 Risk of bias of invidiual studies.docx]

**Supplementary material: Risk of bias for each individual study**
