# Supplementary material for: Placebo effects of caffeine on sport performance: a systematic review and meta-analysis
Source: J Int Soc Sports Nutr. 2026 Oct 20;23(1):2747142. doi: 10.1080/15502783.2026.2747142 (PMC13686187; doi:10.1080/15502783.2026.2747142)
Supplement: Supplementary file 1 [file RSSN_A_2747142_SM2207.zip › 9 GRADE.docx]

| **Supplementary table. GRADE for all analyses comparing placebo caffeine to baseline** | | | | | | |  |  |  |  |
| --- | --- | --- | --- | --- | --- | --- | --- | --- | --- | --- |
| **Certainty assessment** | | | | | | | | **Effect** | **Certainty** | |
| *No of studies* | *Risk of bias* | *Inconsistency* | *Indirectness* | *Imprecision* | *Publication bias* | *Other considerations* | *N participants* | *SMD (95% CI)* |  |  |
| **Main analysis: Placebo caffeine on study primary outcomes** | | | | | | | | | | |
| 17 | Very serious | Not serious | Not serious | Serious | Not serious | None | 197 | 0.45  (0.23 to 0.66) | ⊕⊕○○ | Low |
| **Sub-group analysis: Placebo caffeine on rating of perceived exertion** | | | | | | | | | | |
| 11 | Very serious | Not serious | Not serious | Serious | Not serious | None | 145 | 0.52  (0.26 to 0.78) | ⊕⊕○○ | Low |
| **Sub-group analysis: Placebo caffeine on heart rate** | | | | | | | | | | |
| 6 | Very serious | Very serious | Not serious | Serious | Very serious | None | 64 | 0.21  (-0.13 to 0.55) | ⊕○○○ | Very low |
| **Sub-group analysis: Placebo caffeine on number of repetitions** | | | | | | | | | | |
| 5 | Very serious | Very serious | Not serious | Serious | Very serious | None | 84 | 0.48  (0.06 to 0.90) | ⊕⊕○○ | Very low |
| **Sub-group analysis: Placebo caffeine on power output** | | | | | | | | | | |
| 5 | Very serious | Very serious | Not serious | Serious | Very serious | None | 60 | 0.05  (-0.30 to 0.41) | ⊕○○○ | Very low |
| **Sub-group analysis: Placebo caffeine on velocity** | | | | | | | | | | |
| 4 | Very serious | Not serious | Not serious | Serious | Not serious | None | 25 | -0.15  (-0.51 to 0.20) | ⊕○○○ | Very low |
| **Sub-group analysis: Placebo caffeine on time to complete** | | | | | | | | | | |
| 4 | Very serious | Not serious | Not serious | Serious | Not serious | None | 19 | 0.38  (0.00 to 0.75) | ⊕○○○ | Very low |
| **Sub-group analysis: Placebo caffeine on total weight lifted** | | | | | | | | | | |
| 3 | Very serious | Very serious | Not serious | Serious | Very serious | None | 56 | 0.73  (0.21 to 1.25) | ⊕○○○ | Very low |

| **Supplementary table cont.** | | | | | | | | | | |
| --- | --- | --- | --- | --- | --- | --- | --- | --- | --- | --- |
| **Certainty assessment** | | | | | | | | **Effect** | **Certainty** | |
| *No of studies* | *Risk of bias* | *Inconsistency* | *Indirectness* | *Imprecision* | *Publication bias* | *Other considerations* | *N participants* | *SMD (95% CI)* |  | |
| **Sub-group analysis: Placebo caffeine on Oxygen consumption** | | | | | | | | | | |
| 3 | Very serious | Very serious | Not serious | Serious | Very serious | None | 30 | 0.51  (-0.14 to 1.16) | ⊕○○○ | Very low |
| **Sub-group analysis: Placebo caffeine on lactate** | | | |  |  |  |  |  |  |  |
| 3 | Very serious | Very serious | Not serious | Serious | Very serious | None | 29 | 0.23  (-0.30 to 0.75) | ⊕○○○ | Very low |
| **Sub-group analysis: Placebo caffeine on time to exhaustion** | | | | | | | | | | |
| 2 | Very serious | Not serious | Not serious | Serious | Not serious | None | 17 | 1.07  (0.38 to 1.75) | ⊕○○○ | Very low |
